# Genome-wide profiling of transfer RNAs and their role as novel prognostic markers for breast cancer

**DOI:** 10.1038/srep32843

**Published:** 2016-09-08

**Authors:** Preethi Krishnan, Sunita Ghosh, Bo Wang, Mieke Heyns, Dongping Li, John R. Mackey, Olga Kovalchuk, Sambasivarao Damaraju

**Affiliations:** 1 Department of Laboratory Medicine and Pathology, University of Alberta, Edmonton, Alberta, T6G 2R3, Canada; 2 Department of Oncology, University of Alberta, Edmonton, Alberta, T6G 1Z2, Canada; 3 Cross Cancer Institute, Alberta Health Services, Edmonton, Alberta, T6G 1Z2, Canada; 4 Department of Biological Sciences, University of Lethbridge, Lethbridge, Alberta, T1K 3M4, Canada

## Abstract

Transfer RNAs (tRNAs, key molecules in protein synthesis) have not been investigated as potential prognostic markers in breast cancer (BC), despite early findings of their dysregulation and diagnostic potential. We aim to comprehensively profile tRNAs from breast tissues and to evaluate their role as prognostic markers (Overall Survival, OS and Recurrence Free Survival, RFS). tRNAs were profiled from 11 normal breast and 104 breast tumor tissues using next generation sequencing. We adopted a Case-control (CC) and Case-Only (CO) association study designs. Risk scores constructed from tRNAs were subjected to univariate and multivariate Cox-proportional hazards regression to investigate their prognostic value. Of the 571 tRNAs profiled, 76 were differentially expressed (DE) and three were significant for OS in the CC approach. We identified an additional 11 tRNAs associated with OS and 14 tRNAs as significant for RFS in the CO approach, indicating that CC alone may not capture all discriminatory tRNAs in prognoses. In both the approaches, the risk scores were significant in the multivariate analysis as independent prognostic factors, and patients belonging to high-risk group were associated with poor prognosis. Our results confirmed global up-regulation of tRNAs in BC and identified tRNAs as potential novel prognostic markers for BC.

The discovery that only 2% of the human genome encodes for proteins (the coding portion) and that the remaining 98% (the non-coding portion) harbor sequences with structural, regulatory and functional relevance, dispelled the long-held belief that these sequences should be considered as “junk DNA”^1^. Amongst the non-coding portion of the genome which gets transcribed but not translated, two major classes of RNA exist based on size: long non-coding RNAs (>200 nt) and small non-coding RNAs (sncRNAs <200 nt)^2^. Both the classes of RNA contribute to post-transcriptional level of gene regulation. Several subcategories of sncRNAs exist, including microRNAs (miRNAs), small nucleolar RNAs (snoRNAs), piwi-interacting RNAs (piRNAs) small nuclear RNAs (snRNAs) and transfer RNAs (tRNAs)^3^.

While much of the focus has been on miRNAs^4^, functional significance of other RNAs is less explored in cellular processes and for their potential roles as prognostic markers in cancer. Transfer RNAs (tRNAs) are 73–92 nt long^3^ that play a crucial role in protein synthesis. A total of 625 tRNA genes have been identified so far in the human genome, of which 506 are tRNAs that decode standard amino acids, three are selenocysteine tRNAs, three are suppressor tRNAs, three are tRNAs with undetermined or unknown isotypes and 110 are tRNAs predicted to be pseudogenes^5^.

Apart from playing a role in protein translation, recent discoveries have suggested that tRNAs may play a vital role in activation of protein kinase GCN2^6^, regulation of apoptosis^7^, and protein degradation^8^. Furthermore, processing of the 3′ or 5′ ends of mature or precursor tRNAs have given rise to another class of small RNAs called tRNA derived fragments (tRFs)^9^. Previous studies have demonstrated that tRFs are not degradation by-products but are functional molecules that arise during stress conditions^10^. Relative variations in expression levels of tRFs in tumor cells as compared to normal cells^11^, and their role in silencing gene expression, thereby influencing cell proliferation^9^ or metastasis^12^ implies that they may also contribute to tumorigenesis. Interestingly, there is also evidence indicating that tRFs may possess characteristics of a miRNA, both structurally and functionally (by regulating gene expression)^13^. tRFs have also recently showed promise as prognostic markers for prostate cancer^14^, thus expanding the repertoire of tRNA functions, but their clinical relevance to BC remains unexplored. While miRNAs are known to interact with mRNAs directly and mediate gene expression regulation^15^,^16^, recent evidences have demonstrated the indirect contribution of tRNAs to post-transcriptional gene expression regulation. For instance, Maute *et al*., have identified a functionally active tRNA derived microRNA (miRNA) that represses the expression of protein coding gene by means of sequence complementarity to mRNAs^13^. tRNAs may also act as a source for another molecule called piwi-interacting RNAs (piRNA)^17^, which are also considered as master regulators of gene expression^18^,^19^. This further expands the functions of tRNAs, warranting the need for a deeper exploration into this class of sncRNA.

Dysregulation of protein synthesis machinery has been observed in several tumor cells and has been found to be one of the major contributors for malignant transformation of cells^20^. Specifically, over expression of RNA polymerase III and its products (including tRNAs) has been observed in breast and ovarian cancers^21^,^22^,^23^. Studies on the consequences of aberrant expression of tRNAs have demonstrated that over-expression of initiator tRNA can drive cell proliferation, resulting in oncogenic transformation^24^. As such, tRNAs are now recognized for their pivotal role in tumorigenesis, though a comprehensive understanding of their diverse roles in the biology of cancer is far from complete.

Despite their discovery in 1956^25^,^26^, not many studies have focused on the comprehensive profiling of tRNAs and explored their potential to serve as biomarkers for cancer. Pavon-Eternod *et al*. were the first to profile tRNAs using a microarray platform, to demonstrate that over expression of tRNAs is a hallmark of breast cancer (BC) and postulated their potential utility as biomarkers for BC^27^. However their significance as prognostic markers for BC remains unexplored to date. In fact, the prognostic potential of tRNAs has not been investigated for any type of cancer. Although there has been a considerable progress in creating personalized treatment strategies for BC patients, based on their ER, PR or Her2 receptor expression status, a subset of patients continue to experience recurrence, leading to mortality. Factors contributing to inter-individual variations in response to treatments and eventual clinical outcomes (Overall Survival, OS; Recurrence Free Survival, RFS) may be ascribed in part, to the heterogeneous nature of breast cancer (in terms of histological and molecular subtypes and morphologies)^28^,^29^. The continuing discoveries of additional molecular subsets of BC (based on deep sequencing of tumor genomes) has called for the identification of novel biomarkers or combinations of biomarkers that may perform better than the traditional markers alone, in terms of prognostication or prediction. These molecular signatures may guide the development of target therapies and in the selection of treatment.

In this study, we hypothesized that relative variations in expression levels of tRNAs contribute to inter-individual differences in disease trajectory and in eventual treatment outcomes. We sequenced small RNA libraries generated from 11 apparently healthy normal breast tissue samples (obtained from reduction mammoplasty surgery) and 104 breast tumor samples with complete clinical information^30^. The specific objectives were to (i) profile and identify differentially expressed (DE) tRNAs, (ii) investigate the role of tRNAs as prognostic markers for BC treatment outcomes (OS and RFS), (iii) validate the signatures in an external dataset, and (iv) investigate the contribution of tRNAs to gene regulation. We confirm that tRNAs are globally up-regulated in BC and report for the first time, the prognostic significance of 27 tRNAs.

## Results

### Global over-expression of tRNAs in BC

**Figure 1:**
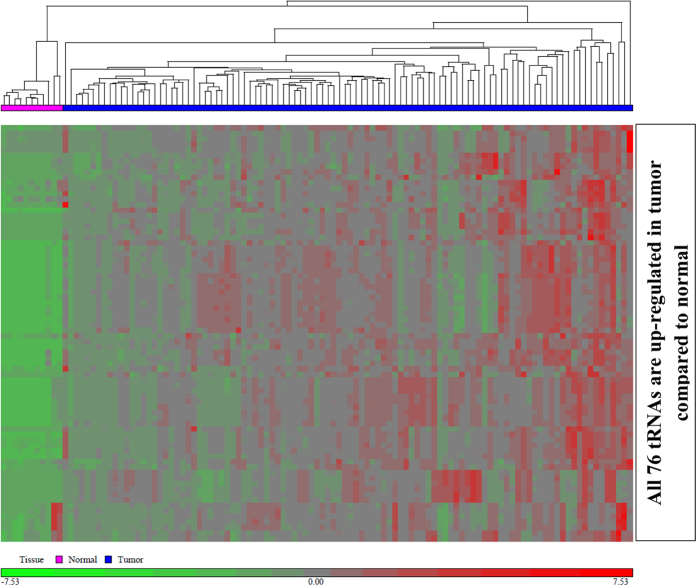
Unsupervised hierarchical clustering using differentially expressed tRNAs. 76 tRNAs were differentially expressed, all of which were up-regulated in tumors and are indicated in red. Euclidean distance was used as a measure of distance and average linkage method was used for linkage analysis. Samples are represented in columns and tRNAs are represented in rows. Blue bar indicates tumor samples.

Details of reads detected in tumor and normal samples have been summarized elsewhere^30^. Briefly, from a total of 164,237,348 and 10,016,964 reads detected from tumor and normal samples respectively, 59% and 51% of the reads were retained after adapter trimming. A total of 25,352,720 reads were mappable to different ncRNA classes (miRNAs, tRNAs, and others). Specifically, 8,247,022 reads were mappable to tRNAs, which accounted to a total of 571 tRNAs across the genome. The raw counts of all tRNAs profiled and the normalized counts of tRNAs adjusted for batch effects (Supplementary Fig. S1) in the CC approach (before and after filtering for read counts) are summarized in Supplementary Tables S1a, S1b and S1c, respectively. One sample was identified as a potential outlier, leaving 102 samples for further analysis. From the 571 tRNAs, 423 were filtered out at the imposed cut-off of 10 read counts in 90% of the samples. The remaining 148 tRNAs were subjected to one-way ANOVA test to identify DE tRNAs. Overall, 76 tRNAs were DE with FC >2 and FDR cut off 0.05 (Supplementary Table S2) and all 76 tRNAs were up-regulated in tumor tissues compared to normal tissues, indicating a global up-regulation of tRNAs in BC. Unsupervised hierarchical clustering (Fig. 1) also demonstrated the ability of tRNAs to discriminate normal samples from tumor samples.

### tRNAs are associated with breast cancer prognosis

Two approaches, Case-control and Case-only (CC and CO) were adopted to identify tRNAs as potential prognostic markers for BC (*vide* methods).

#### Case-control approach

**Figure 2:**
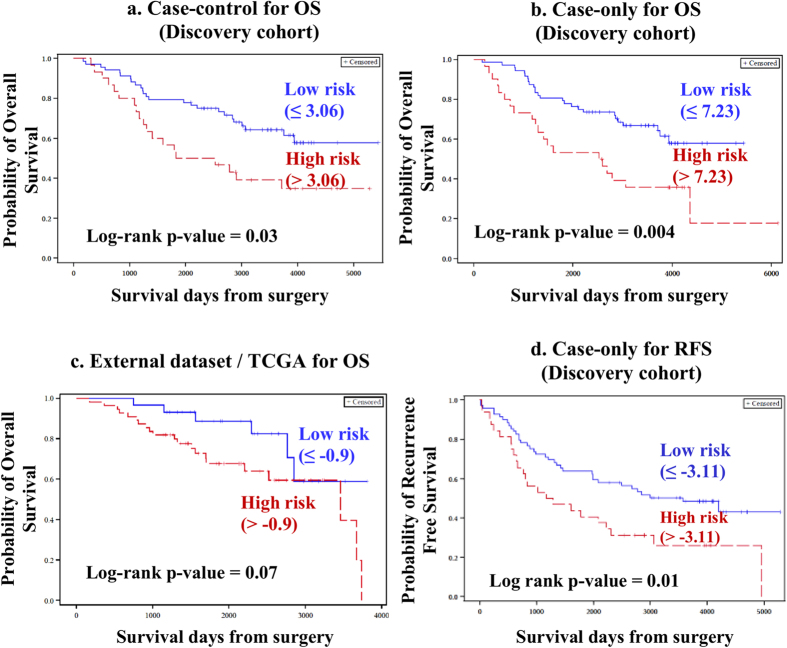
Kaplan-Meier plots for Overall Survival. Probability of OS is plotted over time and the Kaplan-Meier plots indicate that in both Case-control (**a**) and Case-only (**b**) approaches of discovery cohort, patients belonging to high-risk group are associated with poorer OS. A similar trend in survival pattern was observed in the external dataset/TCGA (**c**). (**d**) Probability of RFS is plotted over time and the Kaplan-Meier plots indicate that in the CO approach, patients belonging to high-risk group are associated with poorer RFS.

In the CC approach, survival analysis was restricted to 76 DE tRNAs that were subjected to univariate Cox proportional hazards regression model followed by permutation test. We found three tRNAs (chr6.tRNA5-SerAGA, chr6.tRNA50-SerAGA and chr6.tRNA51-SerTGA) associated with OS that had a permutation p-value ≤ 0.1 (Table 1). These three tRNAs were used to construct a risk score for all cases, and then the cases were dichotomized into two groups based on the ROC estimated cut-off point (3.06). Cases with a risk score ≤3.06 and >3.06 were classified as low-risk and high-risk groups, respectively. Further, the risk score was adjusted for tumor stage and age at diagnosis. High-risk group patients were found to have shorter OS (hazard ratio, HR = 2.68, p = 0.02, CI = 1.19–5.99; Table 2, Fig. 2a). Interestingly, none of the DE tRNAs were found to be associated with RFS.

#### Case-only approach

571 tRNAs were profiled from tumor tissues alone, of which, 216 were retained with ≥10 read counts in at least 90% of tumor samples. The dataset was RPKM normalized and adjusted for batch effects. Raw counts of all the tRNAs and batch adjusted RPKM normalized counts (before and after filtering for read counts) are summarized in Supplementary Tables S1d, S1e and S1f. From the 216 tRNAs (treated as continuous variables), 14 tRNAs were significant for OS in the permutation test, following Univariate Cox analysis (Table 1). The 14 tRNAs included the three tRNAs that were significant in the CC approach. The estimated optimal cut-off point for defining risk groups was 7.23, and patients were stratified into low-risk (≤7.23) and high-risk groups (>7.23). Similar to the CC approach, high-risk group was found to be associated with shorter OS (HR = 2.78, p = 0.0008, CI = 1.53–5.07, Table 2, Fig. 2b). In contrast to the CC approach, 14 tRNAs were found to be significant for RFS (Table 1). A risk score cut-off point of −3.11 separated cases into two survival groups and the high-risk group was found to be associated with shorter RFS (HR = 1.86, p = 0.02, CI = 1.10–3.13, Table 3, Fig. 2d). For both OS and RFS, risk score was found to be significant after adjusting for confounders (tumor stage, grade and age at diagnosis for OS and tumor stage for RFS).

### tRNAs prognostic of overall survival are validated in an external dataset

The batch adjusted normalized counts for tRNAs associated with OS (identified in the CO approach) were extracted from TCGA dataset. Similar to the discovery cohort, risk scores were constructed for every sample and the samples were dichotomized into low and high-risk groups based on the cut-off point (−0.9) estimated using ROC. In the multivariate setting, the risk score was adjusted for tumor stage and age at diagnosis. Statistical significance obtained (p = 0.15) for the risk score indicated a trend similar to the original study (similar direction and magnitude of effect) but did not meet imposed nominal p-value threshold of p < 0.05. Overall, the results from external cohort were supportive of the original study findings that tRNAs are potential prognostic factors; high-risk group was associated with poorer OS (HR = 1.97, p = 0.15, CI = 0.79–4.95, Table 2, Fig. 2c).

### Relative expressions of chr6.tRNA50-SerAGA and chr6.tRNA51-SerTGA are validated using qRT-PCR

**Figure 3:**
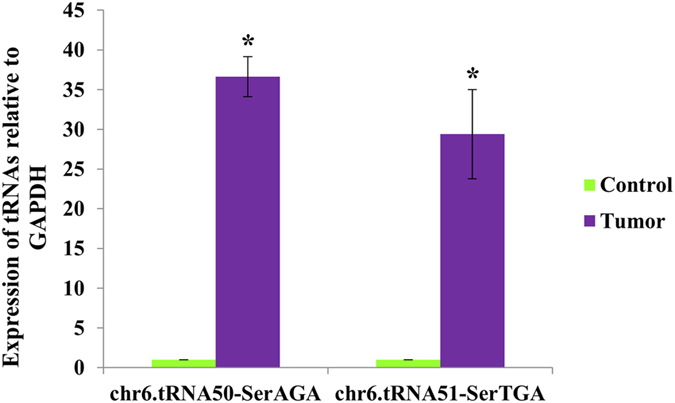
qRT-PCR validation of up-regulated tRNAs. qRT-PCR results of two representative tRNAs confirm their up-regulation in tumors (using GAPDH as the internal normalizer), as indicated by initial NGS experiments. *p < 0.05.

Two representative tRNAs, chr6.tRNA50-SerAGA and chr6.tRNA51-SerTGA, exhibiting a fold change of 2.56 and 2.61, respectively in next generation sequencing (NGS) platform, were validated using qRT-PCR. Both tRNAs were found to be up-regulated in tumors relative to normal tissues in qRT-PCR experiments (Fig. 3), confirming the findings from NGS.

### tRNAs harbor regulatory RNAs and thus contribute to gene regulation

Genomic origins (distinct genes or intergenic or intragenic regions) of tRNAs are not well understood. However, in this study, we observe that a fraction of tRNAs appears to originate from the intronic regions of protein coding or non-protein coding genes. For instance, when we mapped the genomic co-ordinates of the 571 profiled tRNAs to the genomic co-ordinates of messenger RNAs (mRNAs) and long non-coding RNAs (lncRNAs), we observed that ~15% (n = 86) of the tRNAs were embedded within the intronic regions of mRNAs and ~12% (n = 66) were embedded within the introns of lncRNAs (Supplementary Tables S3a and S3b).

Since we now understand that tRNAs may also act as a reservoir for other regulatory RNAs such as microRNAs (miRNAs) and piwi-interacting RNAs (piRNAs), the genomic co-ordinates of the 571 tRNAs were mapped to the genomic co-ordinates of mature miRNAs and piRNAs. 45 tRNAs were observed to harbor piRNAs (Supplementary Table S3c) and one tRNA was found to harbor a miRNA (Supplementary Table S3d). The identified piRNAs and miRNA were subsequently interrogated for differential expression using data generated for our previous studies^19^,^30^. Nine piRNAs (from among the 45 piRNAs annotated to tRNAs) were found to be up-regulated (Table 4). The lone miRNA observed to be within the genomic co-ordinates of a tRNA was not found to be differentially expressed.

Further, to understand the contribution of piRNAs (thereby the tRNAs) to gene regulation, we first identified mRNA targets based on (i) the complementary sequences shared between the piRNAs and the 3′UTR of mRNAs and (ii) the reciprocal expression patterns between piRNAs and mRNAs. Since the nine piRNAs were found to be up-regulated, 2241 genes which were found to be down-regulated in tumor tissues (gene expression dataset), were considered as potential targets for the nine piRNAs. However, when interrogated for sequence complementarity and filtered for stringent alignment and energy scores, 76 targets were identified (Table 4). To understand the functional relevance of the identified targets, gene ontology classification was performed and the identified gene ontology terms (biological processes) are summarized in Table 5. The identified targets were found to be involved in key tumorigenic pathways, including apoptosis and angiogenesis.

## Discussion

This is the first study to profile tRNAs on a genome-wide scale using NGS and to identify their prognostic significance for BC. We profiled 571 tRNAs and found 14 tRNAs each, to be associated with OS and RFS. Amongst these, one tRNA was found to be associated with both OS and RFS. The results also showed similar direction of effect in an external dataset, thereby strengthening the study findings.

tRNAs are among the most abundant molecules present in cells, especially in a metabolically active disease setting such as cancer, indicating higher rate of protein synthesis in these cells^31^. Despite their abundance, they have received less attention as biomarkers mainly due to the complexities involved in developing a profiling platform^32^. The extensive modifications that a tRNA undergoes during maturation and the complicated structure of mature tRNA have deterred the development of a profiling platform as these structural intricacies interfere with reverse transcription and hybridization protocols^32^,^33^. In 2006, however, Dittmar *et al*. developed a microarray method^32^ to profile tRNAs on a genome-wide scale. These microarray protocols could also distinguish between tRNA isoacceptors. Although this is a major leap in the field of tRNA profiling, the method requires custom-made arrays (which may not tend to be cost-effective for large scale profiling experiments) and has limited dynamic range for quantification^34^. However, a recent report by Meng *et al*. confirmed that a wider range of molecules such as the class of sncRNAs, including tRNAs can be profiled using NGS and from clinically archived specimens, preserved as FF or FFPE tissue blocks; forming the rationale for our choice of using NGS platform for tRNA profiling^35^.

We have utilized 104 BC samples with complete clinical annotation, the highest number of samples sequenced for tRNA studies so far. With the advent of NGS, capturing the entire tRNA profile and distinguishing between tRNA isoacceptors is finally possible. We have profiled a total of 571 tRNAs (including isoacceptors), the largest dataset explored for tRNAs so far. Till date, in the human genome, 625 tRNA genes (including pseudogenes) have been identified. tRNAs predominantly arise from chromosome 6 (n = 175), followed by chromosome 1 (n = 137). In our dataset of 571 tRNAs, we have also observed a similar pattern of distribution (Supplementary Table S4), with 170 tRNAs arising from chromosome 6 and 132 from chromosome 1, indicating an unbiased genome-wide capture of tRNAs using the NGS platform.

Our study provides proof of principle experiments in support of the idea that a comprehensive tumor profiling of tRNAs will offer much-needed insights in to new biomarkers for BC prognosis. The two approaches used in our study, CC and CO (do not depend on controls used), are widely accepted means to identify markers of prognostic significance. Although it is not common to adopt both approaches in a single study, we have attempted both the approaches to compare and understand their similarities and differences, in terms of number of signatures and/or the unique or common signatures captured. As anticipated, we identified higher number of prognostically significant tRNAs in the CO approach since the number of tRNAs interrogated for survival analysis was higher. In the case of OS, three tRNAs were found to be significant in CC approach, while 14 tRNAs were identified in CO approach. No tRNA was associated with RFS in CC approach, whereas 14 tRNAs were found to be significant from a CO approach. Therefore, adopting a CO approach not only offers a larger dataset to probe for markers but is also a better option to understand the importance of molecules which would have otherwise been missed in a CC approach that focusses only on DE tRNAs.

We adopted a filtering criterion that enabled us to identify tRNAs present in high amounts, and in most, if not, all of the samples (highly expressed and most frequently expressed). This is one way to improve the chance of reproducibility of the obtained signatures. Indeed, all the 14 tRNAs significant for OS had read counts ≥10 in at least 90% of the samples (except one, which had ≥10 read counts in 87% of the samples) in the external/TCGA dataset, and therefore the overall expression levels were comparable to the discovery dataset. Results of survival analysis from TCGA dataset showed a similar direction of effect; patients belonging to high-risk group were associated with poorer OS, validating our findings from the discovery cohort. The risk score, however, did not reach statistical significance due to the limited sample size and number of events (death) in the cohort, a finding consistent in independent biomarker studies when TCGA dataset is considered^30^. Recurrence events reported for the TCGA dataset are lower than OS and hence the data was not amenable for RFS analysis.

To build a model for multivariate analysis, we did not include individual tRNA molecules identified from the univariate analysis, instead constructed a composite risk score using these RNAs for the following reasons: (i) a complex interplay of biomolecules exists, where each molecule contributes significantly towards a phenotype; (ii) several of the tRNAs identified are highly correlated (Supplementary Tables S5a and S5b) and the pattern of correlation is more pronounced for tRNA isoacceptors (r = >0.9). While this was expected for isoacceptors, it was also interesting to observe fairly high correlation (r = >0.8) between tRNA genes coding for Ser and Leu, the reason for which is unknown. This problem of collinearity which generally leads to spurious associations^36^ of the variables with the outcomes was also overcome by constructing a risk score, which is usually not affected by correlated variables.

Frequently used methods to estimate the cut-off point for patient stratification into two survival groups are–median cut-off point of the risk score and ROC based cut-off point. While calculating the median is the most commonly adopted method, this cut-off point is arbitrary^37^ and does not take into account the sensitivity and specificity of the estimated cut-off point. Conversely, ROC based estimation considers these and is a more reliable measure for cut-off point estimation^38^. We therefore adopted ROC based estimation to determine our cut-off point for patient stratification. Overall, our study has satisfied the parameters set by REMARK guidelines^39^ for biomarker discovery and validation.

We report 76 DE tRNAs that were up-regulated in breast tumor samples, which independently confirms the findings from Pavon-Eternod *et al*. who also reported an overall up-regulation of tRNAs in breast cancer^27^. In the current study, tRNAs coding for 14 amino acids (Arg, Asn, Asp, Gln, Glu, Gly, His, Leu, Lys, Met, Phe, Ser, Val and SeC(e)) clearly showed high DE (FC > 2 and FDR < 0.05). The global up-regulation of tRNAs may be attributed to the high metabolic activity of the cancer cell requiring higher rates of protein synthesis and tRNAs per se may serve diverse non-canonical roles in the cell. Although this phenomenon (global up-regulation of tRNAs) is observed for BC, and similar patterns of expression in other cancer tissues may strengthen this premise.

In the study by Pavon-Eternod, the authors also pointed out the differences in tRNA isoacceptor levels, correlating with the codon preferences of genes involved in tumorigenesis. Although we did not focus on codon-isoacceptor correlations, we did observe differences in tRNA isoacceptor levels for specific amino acids (Supplementary Table S6) in our study, which may correlate with the codon preferences of the genes. For instance, tRNA^Arg(TCT)^ and tRNA^Arg(CCG)^ were expressed in higher amounts when compared to tRNA^Arg(CCT)^. Similarly, tRNA^Leu(CAG)^, tRNA^Leu(CAA)^, and tRNA^Leu(TAA)^ were over expressed, when compared to tRNA^Leu(AAG)^ and tRNA^Leu(TAG)^. tRNAs coding for Gln, Glu and Val also showed preferential expression of certain isoacceptors. In contrast, expression changes of isoacceptors for tRNAs coding for Ser, Gly and Lys remained invariant. In this study, we have observed preferential expression of certain isoacceptors. However further studies are necessary to identify if codon preferences play a role in breast tumorigenesis to explain the observed fold-changes in tRNA isoacceptor levels between normal and tumor tissues. Studies of this kind may further help us understand how tRNAs may directly be involved in breast tumor development.

Recent studies have highlighted the importance of tRNAs as a source for other regulatory RNAs such as piRNAs^17^ and miRNAs^13^, which act as master regulators of gene expression. To this end, we observed that 46 tRNAs may potentially harbor these regulatory RNAs. We also identified that among these 46 regulatory RNAs, nine piRNAs were DE. The nine piRNAs were predicted to target 76 mRNA targets in breast tissues (Table 4). Since these targets are tissue specific, the study premise shows the strength in utilising the transcriptome datasets to serve as a proxy for functional validations of regulatory RNAs. Gene ontology classifications of these targets were enriched for key tumorigenic pathways such as angiogenesis, apoptosis and stem cell maintenance (Table 5).

Although we have identified a potential indirect role of tRNAs in breast tumorigenesis, yet we need to confirm if these tRNAs are indeed giving rise to these regulatory RNAs or if a portion of tRNA merely share sequence similarity to piRNAs. We addressed this question by examining the co-expression of tRNAs and the embedded piRNAs in breast tissues. We report that all nine piRNAs and their corresponding host tRNAs were up-regulated in breast tumor tissues (Table 4). Further experiments are needed to support the piRNA origins to tRNAs. These includes (i) the studies to correlate piRNA and host genes showing similar direction of expression (ii) demonstrate interactions of piRNAs with PIWI proteins, which are the drivers of piRNA biogenesis, (iii) demonstrate direct interaction between the piRNAs and the identified mRNA targets through luciferase expression systems, and (iv) assess potential functions in cellular activities (apoptosis, cell migration, cell proliferation etc) using cell based assays.

Despite tRNAs being one of the oldest molecules discovered, there has been a paucity of data in this field, mainly due to challenges faced in global profiling of tRNAs; constraints to experimentally manipulate the relative expressions in model systems and lastly, limitations in ascribing the phenotypic influence of specific changes in expression levels to cell characteristics and functions^40^. The standard protocol for NGS experiments has helped us uncover the potential of several sncRNAs such as piRNAs^18^ and miRNAs^30^,^41^. tRNAs, however posed challenges even in high-throughput sequencing platforms, mainly due to their compact tertiary structure and the presence of post-transcriptional modifications. This limits the adapter binding efficiency and reverse transcription, both of which are needed to generate libraries and to perform sequencing experiments^42^,^43^, resulting in the generation of truncated sequences from a large subset of tRNAs^44^. Despite this difficulty in tRNA sequencing, we observed a higher number of reads aligning to tRNAs (n = 8,247,022), when compared to piRNAs (n = 4,207,022), snoRNAs (n = 1,610,928) and snRNAs (n = 435,276). However, we observed that the number of tRNA reads were only secondary to miRNAs (n = 25,003,223), which may perhaps be due to the challenges involved in sequencing tRNAs.

Illumina sequencing protocols are still emerging to overcome the inherent limitations described above. We expect a higher number of reads than reported, if the sequencing limitations are overcome. Also, the small RNA sequencing protocol using TruSeq Small RNA library preparation kit has been designed to capture RNAs possessing 5′ phosphate and 3′ hydroxyl group. Ligation of adapters occurs at the ends of RNAs that possess these modification but these adapters can also ligate to other RNAs, albeit inefficiently, which may therefore contribute to the lower abundance of reads mapping to tRNAs. Also, given the read length restriction adopted in our sequencing analysis (17–27 nt), it may not have been feasible to capture full length tRNAs. Therefore the reads captured in this study may likely be the fragments of tRNAs but it is not certain if these fragments are representative of mature full length tRNAs or if they represent actual physiological products (identified as tRFs). Nevertheless, we and others^45^ have confirmed that tRNA sequences can be accurately captured using small RNA sequencing and the reads captured from our study represent the known tRNAs identified and annotated to-date across all chromosomes (Supplementary Table S4).

This is the first study to comprehensively profile tRNAs using NGS and to understand their contribution to BC prognosis. Despite the technical challenges involved in sequencing tRNAs, we have demonstrated a near complete capture of all the annotated tRNAs using the data from the adopted NGS platform. We also found that tRNAs may emerge as promising prognostic biomarkers for BC and an observation of the same pattern of its association with BC prognosis in an external dataset, reaffirms the initial study findings. However, it remains to be seen if these tRNA molecules may perform better as stand-alone biomarkers or if these can complement the existing prognostic markers for BC. Confirmation of the processing of tRNAs to other regulatory RNAs may add a new dimension to the existing knowledge on tRNAs, which may also be beneficial for developing novel therapeutics. We believe that the findings from the current study will encourage more researchers to contribute in delineating the finer molecular mechanisms. Although much remains to be ascertained regarding the various aspects of tRNAs, deeper exploration into this class of RNAs may help us better appreciate the hitherto unexplored biological consequences of these RNAs.

## Methods

### Clinical samples for the discovery cohort

Based on the sample size calculations, as described in our previous miRNA profiling study^30^, it was estimated that at least eight each of normal and tumor tissues (cases and controls) are required to conduct a study with α = 0.05, β = 80% and fold difference of 2 or more. Eleven normal Flash Frozen (FF) breast tissue samples (obtained from reduction mammoplasty surgery) and 104 breast tumor samples preserved as Formalin Fixed Paraffin embedded tissues (FFPE) were accessed from Alberta Cancer Research Biobank/Canadian Breast Cancer Foundation tumor bank (http://www.acrb.ca/). Patient clinical characteristics i.e., tumor histological and molecular profiles, treatments and outcomes of the patients chosen for the study are described elsewhere^30^. Briefly, median age of the BC patients was 50 (Range: 24–79) years and median follow-up period until January 2015 was 2927.5 (Range: 170–6125) days from the date of diagnosis (between years 1998 and 2008). Of the 104 BC patients, 46 patients died and cancer recurred for 61 patients. All the tumor samples exhibited ≥70% tumor cellularity. Written informed consent was obtained from all the patients. The study protocol was approved by the local Institutional Research Ethics Committee (Health Research Ethics Board of Alberta-Cancer Committee). The data generation and analysis from human biological materials were carried out as per the guidelines and regulations set forth by the institution and the ethics board.

### Total RNA isolation

TRIzol and Qiagen RNeasy kit were used for isolating total RNA from normal samples and RecoverAll Total Nucleic Acid Isolation kit (Life Technologies) was used for isolation of total RNA from FFPE blocks. Bioanalyzer 2100 and RNA Nano Chips (Agilent Technologies, Santa Clara, CA) were used for estimating RNA quantity and quality respectively.

### Next generation sequencing (NGS) of small RNA libraries

We utilized services from PlantBiosis Ltd (Lethbridge, Alberta, Canada; (http://www.plantbiosis.com/) for NGS and pertinent protocols were described in detail in our previous study^30^. Briefly, small RNA libraries were prepared for all the samples according to manufacturers’ instructions and were sequenced on Illumina Genome analyzer IIx with 36 cycles single end protocol. CASAVA 1.8.2 was used for base calling and demultiplexing and cutadapt software (https://cutadapt.readthedocs.org/en/stable/)^46^ was used for trimming the adapters. One tumor sample was removed from further analysis due to poor quality score, leaving 103 tumor samples for further analysis. The trimmed sequences were aligned to hg19 genomic assembly using Bowtie^47^, saved as.sam files and converted to memory efficient.bam files. Sequencing files, saved as.bam files are deposited in Gene Expression Omnibus (GEO accession ID GSE68085).

### Identification of differentially expressed tRNAs

Partek Genomics Suite 6.6 (PGS, Partek^®^ Genomics Suite software, version 6.6 beta, Copyright^©^ 2009 Partek Inc., St. Louis, MO, USA) was used for sequencing data analysis. The aligned reads were mapped to UCSC (http://gtrnadb.ucsc.edu) to obtain tRNAs^48^. All the statistical analysis protocols were done as described earlier^19^,^30^. Briefly, tRNAs with raw read counts ≥10 in at least 90% of the samples (tumor and normal inclusive) were retained for further analysis. We normalized the dataset using Reads per kilobase per million (RPKM) method^49^, adjusted the dataset for batch effects (ANOVA model) to offset potential variations due to batched sequencing of samples, and removed any potential sample outliers. The batch effects corrected, filtered and normalized dataset was used for identifying DE tRNAs with a fold change (FC) > 2.0 and a False Discovery Rate (FDR) of 0.05.

### Identification of tRNA signatures as potential prognostic factors for BC

The overall workflow of the study is similar to that adopted for our previous NGS data analysis for identifying miRNAs and piRNAs as prognostic factors for BC^19^,^30^. Two approaches were adopted to identify prognostic signatures – Case-control (CC)^50^ and Case-only (CO) approach^51^. The only difference between the two approaches lies in the tRNA selection procedure for survival analysis. For survival analysis, only DE tRNAs were considered in the CC approach (a method widely adopted by researchers), whereas in the CO approach all the tRNAs retained after filtering (minimum 10 read counts in at least 90% of the tumor samples) were considered, to allow an unbiased selection of markers without self-imposed cut-offs (a method increasingly gaining attention)^30^,^51^. R statistical program and SAS (SAS institute Inc., Cary, NC) version 9.3 were used for downstream statistical analysis. The selected list of tRNAs (considered as continuous variables) from both the approaches were subjected to Univariate Cox proportional hazards regression model with permutation test (n = 10,000) using ‘glmperm’ package in R statistical program. OS and RFS were defined as the time period from the date of surgery until an event occurred (death and relapse, respectively). In both the approaches, tRNAs with a permutation p-value ≤ 0.1 were selected for constructing risk scores for OS and RFS separately. Risk score is a sum of products obtained from multiplying the expression values of each tRNA with its corresponding co-efficient^51^. Cases were dichotomized into low- and high-risk groups based on the risk score cut-off point estimated using Receiver Operating Characteristics (ROC) Curve. Three different cut-off points were estimated – one for CC approach (OS) and two for CO approach (OS and RFS). Multivariate Cox proportional hazards regression model was used to assess if the obtained risk scores (considered as dichotomous variable) were independent prognostic factors after accounting for variables – age at diagnosis (continuous variable), tumor stage (I, II vs III, IV), tumor grade (high vs. low) and TNBC status (Triple Negative vs. Luminal), where applicable. Luminal A, Luminal B and Luminal HER2 were grouped as Luminal. Kaplan-Meier method and log-rank test were used for estimating the median survival function and for comparing the survival curves of the two survival groups (low- and high-risk), respectively. Results for univariate and multivariate analyses are reported along with Hazard ratio (HR) and corresponding 95% confidence interval (CI). P-value of < 0.05 was considered for statistical significance.

### Independent validation of the tRNA prognostic signatures in external data

Samples for external validation were accessed from TCGA project. The 1,088 BC cases available in TCGA dataset were stringently filtered for gender, histological and molecular subtypes, and available clinical information with a follow up period >3 years^30^, to generate a dataset comparable to our discovery cohort. A total of 84 samples with 27 events (deaths), sequenced using Illumina HiSeq, remained after filtering and were used for further analysis using PGS. Similar to the discovery dataset, TCGA dataset was normalized using RPKM method and was adjusted for Batch ID, plate ID and Tissue source site. This dataset was used for obtaining normalized counts of the tRNAs significant for OS (identified in the discovery set). tRNAs associated with RFS was not considered for validation in TCGA dataset as the information on recurrence was limited, if not absent. A new risk score was constructed and an optimal cut-off point was determined for the external dataset, for reasons elucidated (i.e., different sequencing platforms) in our previous study^30^. The risk score was subsequently assessed for its significance in univariate and multivariate Cox proportional hazards regression model (adjusting for tumor stage, age at diagnosis and TNBC status).

### Cross platform concordance to validate expression of select tRNAs

Total RNA isolated from either FF tissue (normal) or from FFPE blocks was subjected to qRT-PCR using iScript Select cDNA Synthesis Kit (Bio-Rad) and SsoFast EvaGreen Supermix (Bio-Rad) according to manufacturers’ instructions. Two tRNAs showing prognostic significance and a FC of >2.0 were chosen for validation using total RNA from nine normal samples and 44 tumor samples. These samples were also used for sequencing experiment. Random primers were used for reverse transcription. Primers for analyzing chr6.tRNA50-SerAGA and chr6.tRNA51-SerTGA were designed with Primer3 software. The sequence of the primer pairs are as follows:

chr6.tRNA50-SerAGA -F: 5′-TAGTCGTGGCCGAGTGGTTA-3′,

chr6.tRNA50-SerAGA -R: 5′-GGAAACCCCAATGGATTTCTA-3′; and for

chr6.tRNA51-SerTGA -F: 5′-TAGTCGTGGCCGAGTGGTTA-3′,

chr6.tRNA51-SerTGA -R: 5′-GAAACCCCAATGGATTTCAA-3′. GAPDH served as the loading control. Primers for analyzing GAPDH by qRT-PCR are described elsewhere^52^. All experiments for qRT-PCR were done in triplicates, data was analyzed using the 2^−ΔΔCt^ method^53^, and results are shown as fold induction of tRNAs.

### Genomic distribution of tRNAs, identification of regulatory RNAs embedded within tRNAs and their roles in gene regulation

With the objective of identifying the possible sites of origin of tRNAs, we overlapped the genomic co-ordinates of all the tRNAs profiled (n = 571) with the genomic co-ordinates of mRNAs and lncRNAs using PGS.

Previous studies have reported that tRNAs may also act as reservoirs for other regulatory RNAs such as miRNAs and piRNAs^13^,^17^. Therefore, the genomic co-ordinates of all 571 tRNAs were overlapped with the genomic co-ordinates of mature miRNAs and piRNAs. Since miRNAs and piRNAs are considered as master regulators of gene expression, potential mRNA targets were identified from gene (mRNA) expression dataset that was available in house (GEO accession ID: GSE22820)^54^. The dataset included 10 normal breast tissues (obtained from reduction mammoplasty) and 141 breast tumor tissues and PGS v 6.6 was used for all the analysis. Raw data was quantile normalized and log2 transformed, and mRNAs exhibiting FC ≥ 2.0 and FDR < 0.05 were identified as DE using ANOVA.

mRNA targets for piRNAs embedded within tRNAs were identified using miRanda v 3.3a. The piRNAs identified to be within the tRNAs were found to be up-regulated in tumor tissues, relative to normal breast tissues^19^. Therefore, fasta sequences of the 3′UTRs of all the down-regulated genes were downloaded from Ensembl database (GRCh37)^55^ and fasta sequences for piRNAs (which were all up-regulated in the study) were downloaded from piRNA bank (hg 19)^56^,. mRNA-piRNAs pairs showing sequence complementarity, with alignment score ≥170 and energy score ≤−20 kcal/mol were identified. The targets thus identified were interrogated for gene ontology classifications to gain functional significance. Gene ontology classification was performed using PGS and gene ontology terms (biological process) showing enrichment score ≥1.3 and a p-value ≤ 0.05 were considered.

## Additional Information

**How to cite this article**: Krishnan, P. *et al*. Genome-wide profiling of transfer RNAs and their role as novel prognostic markers for breast cancer. *Sci. Rep.* **6**, 32843; doi: 10.1038/srep32843 (2016).

## Supplementary Material

Supplementary Tables and Figures

Supplementary Table S1

Supplementary Table S2

Supplementary Table S3

Supplementary Table S4

## Figures and Tables

**Table 1:** tRNAs significant after permutation test.

Overall Survival			Recurrence Free Survival		
tRNA ID	Univariate Cox p-value	Permuted p-value	tRNA ID	Univariate Cox p-value	Permuted p-value
Chr6.tRNA166-AlaAGC	0.02	0.04	Chr6.tRNA166-AlaAGC	0.03	0.03
Chr17.tRNA10-GlyTCC	0.04	0.05	Chr1.tRNA80-GluCTC	0.05	0.04
Chr6.tRNA147-SerAGA	0.04	0.06	Chr1.tRNA77-GluCTC	0.05	0.04
Chr6.tRNA145-SerAGA	0.04	0.06	Chr6.tRNA87-GluCTC	0.07	0.06
**Chr6.tRNA5-SerAGA**	**0.06**	**0.07**	Chr1.tRNA74-GluCTC	0.07	0.06
Chr16.tRNA2-ArgCCT	0.04	0.08	Chr1.tRNA71-GluCTC	0.07	0.06
**Chr6.tRNA50-SerAGA**	**0.07**	**0.09**	Chr1.tRNA59-GluCTC	0.08	0.06
Chr12.tRNA8-AlaTGC	0.08	0.09	Chr6.tRNA77-GluCTC	0.08	0.07
Chr6.tRNA148-SerTGA	0.07	0.09	Chr1.tRNA118-HisGTG	0.1	0.08
Chr6.tRNA172-SerTGA	0.07	0.09	Chr6.tRNA152-ValCAC	0.13	0.08
Chr6.tRNA143-LysTTT	0.06	0.09	Chr1.tRNA116-GluCTC	0.11	0.09
Chr14.tRNA2-LeuTAG	0.07	0.09	Chr2.tRNA19-GlyGCC	0.12	0.09
**Chr6.tRNA51-SerTGA**	**0.08**	**0.09**	Chr6.tRNA128-GlyGCC	0.11	0.09
Chr9.tRNA4-ArgTCT	0.06	0.10	Chr1.tRNA133-GlyCCC	0.12	0.09

Two approaches were adopted to select the set of tRNAs for survival analysis. In the CC approach and CO approach, 76 DE tRNAs and 216 tRNAs (retained after filtering for read counts) were selected for Univariate Cox proportional hazards regression model, followed by permutation test. Left panel of the table includes OS significant tRNAs (permuted p-value ≤ 0.1) from both the approaches (n = 3 in CC and n = 14 in CO). The CO approach also included the tRNAs that were significant in the CC approach, which are indicated in bold. Right panel of the table includes tRNAs significant for RFS in CO approach. None of the tRNAs were identified as associated with RFS from the CC approach.

**Table 2:** Univariate and Multivariate results for Overall Survival.

Parameter	Case-control approach				Case-only approach				External dataset (TCGA)			
	Univariate analysis		Multivariate analysis		Univariate analysis		Multivariate analysis		Univariate analysis		Multivariate analysis	
	HR (95% CI)	p-value	HR (95% CI)	p-value	HR (95% CI)	p-value	HR (95% CI)	p-value	HR (95% CI)	p-value	HR (95% CI)	p-value
Risk score	2.39 (1.07–5.33)	0.03	2.68 (1.19–5.99)	0.02	2.33 (1.29–4.18)	0.01	2.78 (1.53–5.07)	0.001	2.28 (0.92–5.66)	0.08	1.97 (0.79–4.95)	0.15
Tumor stage	0.40 (0.21–0.78)	0.01	0.50 (0.25–1.01)	0.05	0.40 (0.21–0.78)	0.01	0.46 (0.23–0.93)	0.03	0.32 (0.13–0.78)	0.01	0.29 (0.11–0.74)	0.009
Tumor grade	2.01 (1.04–3.89)	0.04			2.01 (1.04–3.89)	0.04	2.49 (1.26–4.93)	0.01	—	—	—	—
Age at diagnosis	1.06 (1.02–1.09)	0.001	1.05 (1.02–1.09)	0.002	1.06 (1.02–1.09)	0.001	1.05 (1.02–1.09)	0.001	1.03 (1.003–1.06)	0.03	1.03 (1.01–1.06)	0.02
TNBC status	0.99 (0.50–1.95)	0.98			0.99 (0.50–1.95)	0.98			0.63 (0.19–2.12)	0.46		

HR = Hazard Ratio; CI = Confidence Interval; TNBC = Triple Negative Breast Cancer.

Risk scores were constructed for the discovery cohort from the three and 14 tRNAs (significant for OS) identified from CC (left panel) and CO (middle panel), respectively. tRNAs significant for OS were validated in TCGA/external dataset (right panel). Patients were dichotomized into low and high-risk groups based on ROC estimated cut-off point. Risk score was significant for univariate and multivariate Cox analysis and patients belonging to high-risk group were associated with poorer OS (HR > 1).

**Table 3:** Univariate and Multivariate results for Recurrence Free Survival: Case-only approach (Discovery cohort).

Parameter	Univariate analysis		Multivariate analysis	
	HR (95% CI)	p-value	HR (95% CI)	p-value
Risk score	1.89 (1.13–3.19)	0.02	1.86 (1.10–3.13)	0.02
Tumor stage	0.38 (0.20–0.71)	0.003	0.39 (0.21–0.73)	0.003
Tumor grade	1.58 (0.92–2.74)	0.10		
Age at diagnosis	1.02 (0.99–1.05)	0.21		
TNBC status	0.84 (0.45–1.55)	0.58		

HR = Hazard Ratio; CI = Confidence Interval; TNBC = Triple Negative Breast Cancer.

Risk scores were constructed from the 14 tRNAs that were significant for RFS in the CO approach. Patients were dichotomized into low and high risk groups based on ROC estimated cut-off point. Univariate Cox analysis was run for risk score and other clinical variables (included in the table). Risk score was further adjusted for potential confounders and was found to be significant (p < 0.05). Patients belonging to high-risk group were associated with poorer RFS (HR > 1).

**Table 4:** List of gene targets identified by piRNAs embedded within tRNAs.

tRNA ID (Fold change)	piRNA ID (Fold change)	mRNA targets (Down-regulated)
chr1.trna68-GlyGCC (1.88)	hsa_piR_000291 (1.71)	TNKS, ZC3H6, ZHX3
chr2.trna19-GlyGCC (1.99)	hsa_piR_000765 (1.85)	SCN2B, SH3TC2, SEMA6D, SLC16A4, SYNPO, TMTC1, TSHZ2, TIFA, TRPM3, WFIKNN2, ZSCAN12, UBQLNL, APCDD1, CNR1, CES2
chr6.trna13-LysCTT (14.79)	hsa_piR_000794 (1.94)	RRAD, SLC2A4, SEMA3E, RPL18, ZNF366, WSCD1, B3GAT1, CACNA1B, CES2
chr6.trna5-SerAGA (2.46)	hsa_piR_015249 (2.42)	NONE
chr6.trna87-GluCTC (1.35)	hsa_piR_017716 (1.51)	SEMA3G, SCARA3, SIRPA, RSPO1, SLC23A2, RPS9, SLC34A2, ST8SIA2, TMCC3, TLN2, TNFSF12, TRIM2, TIFA, ZNF395, TXNRD2, VPRBP, ADAM11, ACVR1C, ANGPTL4, ACACB, ALS2CL, APOL4, ALPL, ARID5A, ATP13A4, ACSM1, CLEC4M, CLIP3, CCDC120, CCDC38
chr19.trna8-SeC(e)TCA (18.15)	hsa_piR_019912 (16.64)	SDK2, SYNPO
chr12.trna13-AlaTGC (1.14)	hsa_piR_020485 (1.11)	SLC2A4, SEC63, TMEM87A, USP31, VPS13A, AKR1C1, ABCG5, ALG9
chr2.trna3-AlaAGC (1.87)	hsa_piR_020496 (1.87)	ALG9
chr5.trna15-ValAAC (9.57)	hsa_piR_020829 (9.58)	SCN2B, SACS, RYR1, SNCAIP, WNT5B, ARHGAP26, CAPN6, CD34

45 piRNAs were found to be embedded within tRNAs, of which nine piRNAs were found to be differentially expressed. Since these 9 piRNAs were up-regulated, potential targets were identified from the genes that were down-regulated in breast tumor tissues. A total of 76 gene targets were identified for the 9 piRNAs.

**Table 5:** Gene ontology classification for the piRNA targets.

Gene ontology classification	mRNA targets	piRNAs regulating mRNA target expression
Regulation of angiogenesis	SEMA3E, TNFSF12, ANGPTL4, CD34	hsa_piR_000794, hsa_piR_017716, hsa_piR_020829
Apoptotic nuclear changes	ACVR1C	hsa_piR_017716
Fat cell differentiation	SLC2A4, CLIP3, WNT5B	hsa_piR-000794, hsa_piR_020485, hsa_piR_017716, hsa_piR_020829
Regulation of Wnt signaling pathway	TNKS, APCDD1, RSPO1, WNT5B	hsa_piR_000291, hsa_piR_000765, hsa_piR_017716, hsa_piR_020829
Doxorubicin and Daunorubicin metabolic process	AKR1C1	hsa_piR_020485
Negative regulation of intracellular estrogen receptor signaling pathway	ZNF366	hsa_piR_000794
Progesterone metabolic process	AKR1C1	hsa_piR_020485
Hematopoietic stem cell proliferation	CD34	hsa_piR_020829

Representative gene ontology terms with enrichment score > 1.3 and p-value < 0.05 are listed. Each row in columns two and three represent the mRNA targets involved in the functions and the corresponding piRNAs predicted to bind to these targets.
