# Supplementary material for: Genome-wide profiling of transfer RNAs and their role as novel prognostic markers for breast cancer
Source: Sci Rep. 2016 Sep 8;6:32843. doi: 10.1038/srep32843 (PMC5015097; doi:10.1038/srep32843)
Supplement: Supplementary Tables and Figures [file srep32843-s1.pdf]

Genome wide profiling of transfer RNAs and their role as novel prognostic markers for breast cancer

Preethi Krishnan, Sunita Ghosh, Bo Wang, Mieke Heyns, Dongping Li, John R Mackey, Olga Kovalchuk, Sambasivarao Damaraju

SUPPLEMENTARY TABLES AND FIGURES

Supplementary Fig S1: Batch effects correction using ANOVA model

a. Data before batch correction

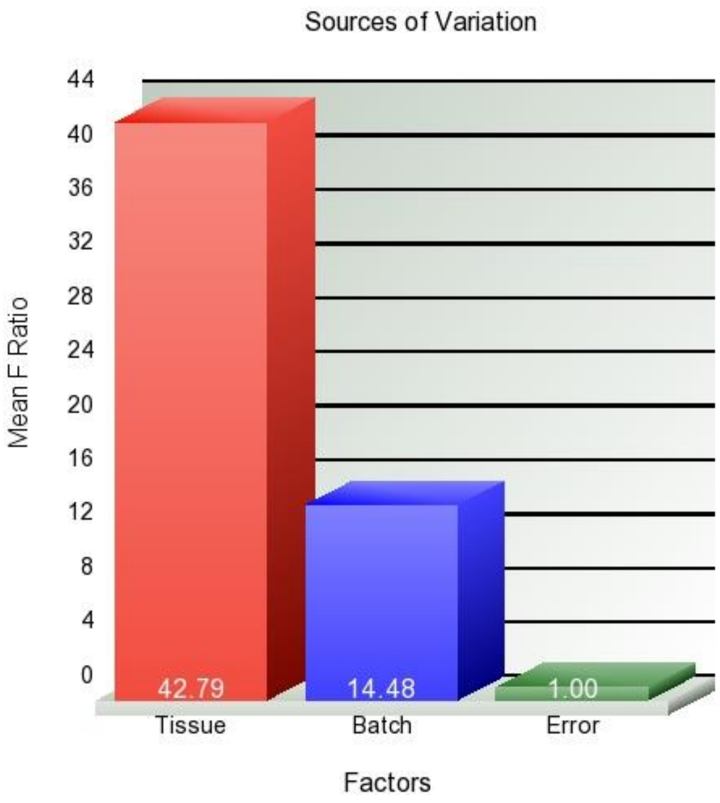

b. Data after batch correction

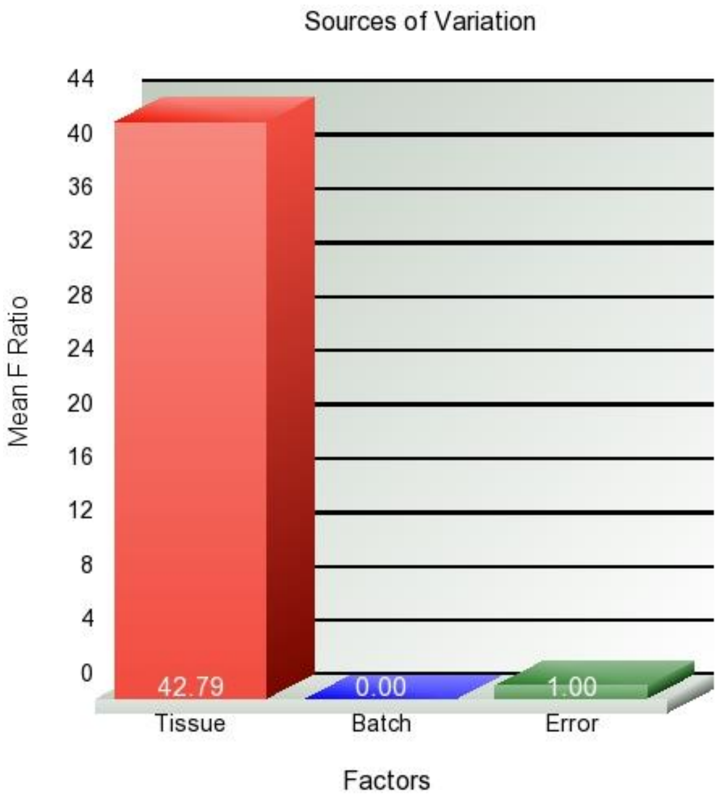

Tissue = Normal and Tumor tissue; Batch = Different batches in which the samples were sequenced

Supplementary Fig S1: Samples used for the study were sequenced in different batches and this tends to generate batch effects, which is a technical variation. Figure S1a represents the different sources of variation in the dataset and any source of technical variation with a mean F ratio above the mean F ratio of error bar will be corrected for. Since batch is a technical variation and tissue (which includes normal and tumor tissue) is a biological variation, the dataset was not adjusted for tissue but was adjusted for batch. Figure S1b on the right represents the sources of variation graph after adjusting for batch (indicated by mean F ratio of 0).

Supplementary Table S1: Raw counts and normalized counts (See supplementary Table S1.xls)

Supplementary Table S2: List of differentially expressed tRNAs

tRNA ID	p-value	FDR	Fold Change	Direction of fold change
chr1.trna119-LysCTT	3.97E-15	1.67E-14	10.02	Up-regulated in tumor
chr1.trna16-HisGTG	4.06E-03	4.54E-03	100.75	Up-regulated in tumor
chr1.trna21-HisGTG	4.82E-03	5.16E-03	35.22	Up-regulated in tumor
chr1.trna26-AsnGTT	1.26E-10	2.45E-10	2.51	Up-regulated in tumor
chr1.trna34-LeuCAG	1.29E-14	4.91E-14	2.69	Up-regulated in tumor
chr1.trna36-LeuCAG	8.97E-17	1.70E-15	2.24	Up-regulated in tumor
chr1.trna38-LeuCAG	1.35E-15	9.14E-15	2.61	Up-regulated in tumor
chr1.trna40-LeuCAG	2.45E-15	1.17E-14	2.36	Up-regulated in tumor
chr1.trna42-LeuCAG	2.18E-15	1.14E-14	2.47	Up-regulated in tumor
chr1.trna47-AsnGTT	2.47E-07	3.61E-07	2.72	Up-regulated in tumor
chr1.trna54-LysTTT	8.15E-13	2.14E-12	3.64	Up-regulated in tumor
chr1.trna58-LeuCAA	5.54E-16	5.29E-15	2.04	Up-regulated in tumor
chr1.trna62-LysTTT	9.85E-13	2.42E-12	4.10	Up-regulated in tumor
chr1.trna67-LeuCAG	3.61E-15	1.61E-14	2.29	Up-regulated in tumor
chr1.trna7-AsnGTT	4.97E-09	9.00E-09	3.15	Up-regulated in tumor
chr1.trna9-ArgTCT	1.99E-07	2.96E-07	4.27	Up-regulated in tumor
chr2.trna27-GlyCCC	9.83E-13	2.42E-12	4.07	Up-regulated in tumor
chr5.trna11-LysCTT	1.09E-05	1.36E-05	7.08	Up-regulated in tumor
chr5.trna15-ValAAC	4.00E-09	7.42E-09	2.07	Up-regulated in tumor
chr5.trna9-LysCTT	1.10E-07	1.77E-07	15.79	Up-regulated in tumor
chr6.trna109-PheGAA	8.92E-06	1.17E-05	10.93	Up-regulated in tumor
chr6.trna114-ArgCCG	3.65E-07	5.14E-07	39.76	Up-regulated in tumor
chr6.trna129-MetCAT	7.54E-08	1.27E-07	11.06	Up-regulated in tumor
chr6.trna13-LysCTT	3.23E-18	8.82E-17	5.71	Up-regulated in tumor
chr6.trna142-MetCAT	3.48E-18	8.82E-17	6.26	Up-regulated in tumor
chr6.trna150-MetCAT	3.18E-18	8.82E-17	4.76	Up-regulated in tumor

chr6.trna169-MetCAT	4.69E-03	5.09E-03	33.56	Up-regulated in tumor
chr6.trna171-MetCAT	6.28E-15	2.51E-14	42.31	Up-regulated in tumor
chr6.trna1-GlnCTG	1.57E-15	9.18E-15	118.90	Up-regulated in tumor
chr6.trna33-HisGTG	1.36E-15	9.14E-15	2.54	Up-regulated in tumor
chr6.trna44-SerAGA	5.84E-05	7.04E-05	32.80	Up-regulated in tumor
chr6.trna45-AspGTC	1.15E-15	9.14E-15	4.06	Up-regulated in tumor
chr6.trna46-SerAGA	4.87E-16	5.29E-15	2.45	Up-regulated in tumor
chr6.trna48-AspGTC	1.44E-15	9.14E-15	9.65	Up-regulated in tumor
chr6.trna49-GlnCTG	1.46E-03	1.70E-03	2.83	Up-regulated in tumor
chr6.trna50-SerAGA	1.52E-12	3.51E-12	17.75	Up-regulated in tumor
chr6.trna51-SerTGA	8.54E-08	1.41E-07	2.84	Up-regulated in tumor
chr6.trna5-SerAGA	8.47E-07	1.15E-06	4.83	Up-regulated in tumor
chr6.trna72-PheGAA	5.86E-12	1.24E-11	3.63	Up-regulated in tumor
chr6.trna73-ArgCCG	9.96E-14	2.91E-13	2.47	Up-regulated in tumor
chr6.trna76-LysTTT	3.06E-07	4.39E-07	7.29	Up-regulated in tumor
chr6.trna7-LeuCAG	5.39E-09	9.52E-09	5215.67	Up-regulated in tumor
chr6.trna83-LeuTAA	1.12E-07	1.77E-07	13.69	Up-regulated in tumor
chr6.trna96-PheGAA	2.37E-10	4.50E-10	2.65	Up-regulated in tumor
chr8.trna10-MetCAT	6.25E-07	8.64E-07	21.58	Up-regulated in tumor
chr11.trna14-LysTTT	1.26E-16	1.91E-15	18.15	Up-regulated in tumor
chr11.trna17-ValTAC	2.70E-03	3.06E-03	2.52	Up-regulated in tumor
chr11.trna5-LysTTT	2.25E-15	1.14E-14	9.69	Up-regulated in tumor
chr12.trna11-PheGAA	1.49E-05	1.82E-05	9.57	Up-regulated in tumor
chr12.trna4-AspGTC	1.36E-12	3.24E-12	17.94	Up-regulated in tumor
chr12.trna5-AspGTC	1.70E-07	2.58E-07	18.72	Up-regulated in tumor
chr13.trna1-PheGAA	1.90E-03	2.18E-03	4.64	Up-regulated in tumor
chr13.trna3-GluTTC	5.04E-03	5.33E-03	8.98	Up-regulated in tumor
chr14.trna13-LysCTT	1.70E-12	3.80E-12	14.78	Up-regulated in tumor
chr15.trna11-GluTTC	1.56E-02	1.62E-02	4.77	Up-regulated in tumor

chr15.trna1-HisGTG	3.09E-02	3.09E-02	4.08	Up-regulated in tumor
chr15.trna2-LysCTT	2.78E-02	2.82E-02	4.66	Up-regulated in tumor
chr16.trna10-LysCTT	1.62E-02	1.66E-02	5.30	Up-regulated in tumor
chr16.trna17-LeuCAG	5.76E-12	1.24E-11	3.78	Up-regulated in tumor
chr16.trna1-ArgCCG	4.47E-03	4.93E-03	114.61	Up-regulated in tumor
chr16.trna22-MetCAT	2.46E-14	7.80E-14	2.31	Up-regulated in tumor
chr16.trna26-LeuCAG	1.01E-05	1.28E-05	12.05	Up-regulated in tumor
chr16.trna32-LysCTT	1.97E-14	7.06E-14	2.42	Up-regulated in tumor
chr16.trna34-GlyCCC	1.01E-05	1.28E-05	11.42	Up-regulated in tumor
chr16.trna7-LysCTT	4.80E-14	1.46E-13	2.94	Up-regulated in tumor
chr17.trna19-ArgTCG	1.42E-13	3.85E-13	2.56	Up-regulated in tumor
chr17.trna23-ArgCCG	2.04E-14	7.06E-14	2.61	Up-regulated in tumor
chr17.trna2-LysTTT	1.14E-13	3.20E-13	2.46	Up-regulated in tumor
chr17.trna3-GlnCTG	1.15E-06	1.54E-06	60.82	Up-regulated in tumor
chr17.trna4-ArgTCT	8.60E-05	1.00E-04	14.89	Up-regulated in tumor
chr18.trna4-LysCTT	8.29E-12	1.66E-11	4.10	Up-regulated in tumor
chr19.trna14-PheGAA	5.56E-16	5.29E-15	2.49	Up-regulated in tumor
chr19.trna1-AsnGTT	2.43E-14	7.80E-14	3.98	Up-regulated in tumor
chr19.trna2-GlyTCC	1.44E-07	2.23E-07	12.94	Up-regulated in tumor
chr19.trna8-SeC(e)TCA	7.72E-12	1.59E-11	3.09	Up-regulated in tumor
chrX.trna4-ValTAC	3.54E-08	6.12E-08	17.17	Up-regulated in tumor

FDR = False Discovery Rate

Supplementary Table S2: 11 normal and 104 tumor samples were sequenced, 571 tRNAs were profiled in the breast tissues with at least 1 read count, of which 148 were retained after filtering. 76 tRNAs (represented in the table) were differentially expressed (ANOVA 1-way) with Fold change > 2.0 and FDR cut off 0.05. All 76 tRNAs were up-regulated.

Supplementary Table S3: Origin of tRNAs and tRNAs as reservoirs for other RNAs (See Supplementary Table S3.xls)

Supplementary Table S4: Distribution of tRNAs

Chromosome	Number of tRNAs identified in human genome	Number of tRNAs identified in our dataset
1	139	132
2	28	22
3	12	10
4	5	4
5	24	21
6	175	170
7	26	25
8	14	9
9	8	6
10	6	3
11	19	16
12	16	14
13	7	7
14	23	22
15	11	10
16	34	33
17	42	38
18	4	4
19	14	12
20	7	4
21	2	1
22	1	1
X	7	6
Y	1	1

Supplementary Table S5: Correlation matrix for OS and RFS tRNAs (See Supplementary Table S5.xls)

Supplementary Table S6: Fold changes of tRNA isoacceptors (See Supplementary Table S6.xls)
